# Physico‐chemical properties, antioxidant activity, and ACE inhibitory activity of protein hydrolysates from wild jujube seed

**DOI:** 10.1111/1750-3841.16157

**Published:** 2022-05-03

**Authors:** Rongxin Han, Shuai Shao, Hongyin Zhang, Hongyu Qi, Fengqin Xiao, Yingxin Shen, Lin Fan, Haidong Wang, Daqing Zhao, Guangzhe Li, Mingming Yan

**Affiliations:** ^1^ Changchun University of Chinese Medicine Changchun China; ^2^ Jilin Provincial Science and Technology Innovation Center of Health Food of Chinese Medicine Changchun University of Chinese Medicine Changchun Jilin China

**Keywords:** ACE inhibitory activity, antioxidant activity, HepG2 cells, hydrolysates, physico‐chemical properties, wild jujube seed protein

## Abstract

**Abstract:**

Wild jujube seed protein (WJSP) as one kind of functional food material has attracted much attention due to its highly nutritive and medicinal value in anti‐inflammatory and improving immunomodulatory ability. However, owing to its large molecular weight and complex structure, biological activities of WJSP were greatly limited and cannot be fully utilized by the human body. Therefore, how to improve the bioavailability of WJSP and develop promising WJSP nutritious materials is a great challenge. In this work, wild jujube seed protein hydrolysates (WJSPHs) were prepared from WJSP via enzymatic hydrolysis method, and their physico‐chemical properties, antioxidant activity, and angiotensin converting enzyme (ACE) inhibitory activity in vitro have been investigated for the first time. SDS–PAGE electrophoresis and size–exclusion chromatographic results indicate that WJSPHs have lower molecular weight distribution (< 5,000 Da) than WJSP. Circular dichroism (CD) spectroscopy and Fourier transform infrared spectroscopy (FTIR) results illustrated that random coil is the main secondary structure of WJSPHs. Antioxidant experiments indicate that WJSPHs exhibit high radicals‐scavenging ability of 1,1‐diphenyl‐2‐picrylhydrazyl (DPPH) radicals (94.60%), 2,2′‐azinobis‐(3‐ethylbenzthiazoline‐6‐sulfonate) (ABTS^+^) radicals (90.84%), superoxide radicals (44.77%), and hydroxyl radicals (47.77%). In vitro, WJSPHs can significantly decrease the accumulation of reactive oxygen species (ROS) and malondialdehyde (MDA), and increase the activity of superoxide dismutase (SOD) and glutathione peroxidase (GSH‐Px) in HepG2 cells. Moreover, ACE activity was found that can be significantly inhibited by WJSPHs (73.02%). Therefore, all previously mentioned results suggest that WJSPHs may be a promising antioxidant food to prevent oxidative‐related diseases in future.

**Practical Application:**

This study shows that WJSPHs exhibit high antioxidant activity and ACE inhibitory activity in vitro, which provide potential application value as antioxidant peptides to prevent oxidative‐related diseases.

## 1 INTRODUCTION

Wild jujube, as a rhamnaceae plant is extensively distributed in north and middle–south region of China. Recently, wild jujube has become a popular functional food material due to its high nutritive and medicinal value in promoting digestion, improving sleep quality, and in anti‐hypertension (Li et al., 2017). These advantages promoted the consumption and development of wild jujube in health products markets in Asia (Guo et al., 2020). The seeds of wild jujube have been widely utilized not only in the clinical application of traditional Chinese medicine over the past few thousand years, but also used for the source of health foods in daily diet, such as tea, porridge, and health drinks. Wild jujube seeds are rich in flavonoids, saponins, alkaloids, and protein, and contain 17 kinds of essential amino acids. Wild jujube seed protein (WJSP) occupies approximately 36% on a dry weight basis of wild jujube seed. Our previous studies found that WJSP can be used as one kind of popular functional food material to promote anti‐inflammatory and immunomodulatory effects (Zhang et al., 2020). However, owing to its large molecular weight and complex structure, biological activities of WJSP, such as antioxidant activity and antifatigue, are greatly limited and cannot be fully utilized by the human body after ingestion. Therefore, how to improve the bioavailability of WJSP and develop promising WJSP nutritious materials is a great challenge (Betancur‐Ancona et al., 2014).

Protein can be effectively converted into peptides via enzymatic hydrolysis method by reducing the size and modifying the charge, to improve its functional characteristics and biological activities without affecting its nutritional value. Nowadays, enzymatic hydrolysis has been widely applied in the structural modification of proteins and the preparation of bioactive peptides (Evangelho et al., 2017; Ghribi et al., 2015; Moure et al., 2006). Previous studies have certified that a series of hydrolysates from plant protein, such as soybean, mung bean, black bean, rice, and millet protein hydrolysates, have shown beneficial health effects such as antioxidant, anti‐hypertensive, antibacterial, antifatigue, and immunomodulatory activities (Liu et al., 2018; Xia et al., 2020; Zheng et al., 2019). Moreover, many researchers demonstrated that the hydrolysates and bioactive polypeptides can be used as nutritional resources in the functional food industry (Agrawal et al., 2020; Chang et al, 2020; Yu et al., 2021).

In recent years, studies on wild jujube seed protein hydrolysates (WJSPHs) have been rarely reported. Ketaki et al. (2016) found that jujube seed protein showed good solubility and possessed interfacial properties, and its papain hydrolysates showed antioxidant activity in vitro. However, the physico‐chemical properties of jujube seed protein hydrolysates were rarely studied. In this study, we determined and obtained the optimal protease by testing the degree of hydrolysis (DH) and 1,1‐diphenyl‐2‐picrylhydrazyl (DPPH) radical‐scavenging ability, and also using sodium dodecyl sulfate‐polyacrylamide gel electrophoresis (SDS–PAGE) and scanning electron microscopy (SEM). Then, WJSPHs were successfully obtained from WJSP by using the optimal protease. This work aimed to analyze the amino acid composition, molecular weight distribution, and structural properties of WJSPHs. Furthermore, this study evaluated the antioxidant activity and angiotensin converting enzyme (ACE) inhibition of WJSPHs in vitro. Consequently, all previously mentioned studies certify the potential application value of WJSPHs in the health food industry.

## 2 MATERIALS AND METHODS

### 2.1 Materials

Wild jujube seeds (*Ziziphus jujuba* Mill.var.*spinosa* (Bunge) HuexH.F. Chou) were obtained from the Jilin Hospital of Traditional Chinese Medicine, and the seeds were originated from Shanxi province of China.

DPPH, 2,2′‐azinobis‐(3‐ethylbenzthiazoline‐6‐sulfonate) (ABTS^+^), and ACE were obtained from Beijing Solarbio Science & Technology Co., Ltd. (Beijing, China). Alcalase, neutrase, papain, and hippuric acid (HA) were obtained from Shanghai Yuanye Bio‐Technology Co., Ltd. (Shanghai, China). 2′, 7′‐ Dichlorodihydrofluorescein diacetate (DCFH‐DA) was purchased from Beyotime Biotechnology Co., Ltd. (Shanghai, China). Malondialdehyde (MDA), glutathione peroxidase (GSH‐Px), and superoxide dismutase (SOD) test kits were purchased from Nanjing Jiancheng Bioengineering Institute (Nanjing, China). Hippuryl‐histidyl‐leucine (HHL) was purchased from Sigma Chemical Company (St. Louis, MO, USA). Other reagents and chemicals were of analytical grade.

### 2.2 Preparation of WJSP and WJSPHs

WJSP was prepared from the wild jujube seeds defatted powder using the methods described by Zhang et al. (2020) with some alterations. Then WJSP was dissolved in deionized water to prepare a 5% protein solution (w/v). Alcalase, neutrase, and papain were subsequently added in the obtained solution at the final enzyme to substrate ratio (E/S) of 2%. The hydrolysis of alcalase, neutrase, and papain were performed at 55℃ and pH 8.0, 50℃ and pH 7.0, and 60℃ and pH 6.0, respectively, and the pH values were maintained by adding 1 mol/L NaOH solution. And the reaction time of enzymolysis was 2 h. The obtained mixture was further heated at 100℃ for 10 min to deactivate the enzyme. After cooling, the pH of obtained mixture was adjusted to 7. The prepared mixture was centrifuged at 8000*g* for 20 min. The collected supernatants were lyophilized, and then stored at −20℃ for further study.

### 2.3 Degree of hydrolysis (DH)

Degree of hydrolysis is described as the percentage of the number of peptide bonds hydrolyzed to the number of total peptide bonds in protein substrate. In this study, DH of WJSPHs was measured by an *o*‐phthaldialdehyde (OPA) method with slightly revisions (Nielsen et al., 2001). About 400 µl of protein hydrolysates solution was mixed with 3 ml OPA solution. The mixed solution was shaken and reacted for 2 min at 25℃, and the absorbance of mixture was determined at 340 nm by a UV–visible spectrophotometer (Shimadzu UV−1800, Tokyo, Japan).

### 2.4 Physico‐chemical characteristics

#### 2.4.1 SDS‐PAGE and Tricine‐SDS‐PAGE electrophoresis

The electrophoretogram of WJSP and WJSPHs was measured according to the previous report (Jiang et al., 2018). Briefly, SDS**–**PAGE electrophoresis used 12.5% separating gel and 5% stacking gel, and Tricine**–**SDS**–**PAGE electrophoresis used 16.5% separating gel, 10% sandwich gel, and 4% stacking gel. The sample solution was blended with loading buffer in same volume, and 10 µl of the mixed solution was added into the gel. SDS**–**PAGE electrophoresis was carried out by Bio‐Rad electrophoresis apparatus, and the samples were run at 70 V for 30 min and subsequently at 140 V for 1 h. Tricine**–**SDS**–**PAGE electrophoresis was carried out by Bio‐Rad electrophoresis apparatus, and all samples were allowed to run at 30 V for 1 h and subsequently at 100 V for 5 h. Finally, the obtained gels were dyed in Coomassie Bright Blue solution and destained in a mixed solution (glacial acetic acid:methanol:water = 1:3:6).

#### 2.4.2 Scanning electron microscopy

The spatial morphology of three protein hydrolysates was observed via SEM on the basis of the methods described by Noman et al. (2020) with slightly modifications. Initially, lyophilized protein hydrolysates were passed through a 100‐mesh sieve and mounted on holders with the double‐faced adhesive. Then, a thickness of 10–20 nm of gold was covered on the samples by using an ion sputter apparatus. Subsequently, the morphology of samples was determined through a scanning electron microscope (Hitachi S−3400N, Hitachi, Japan) at a magnification of × 100 under the accelerating beam voltage of 15.00 kV.

#### 2.4.3 Molecular weight distribution

The molecular weight distribution of WJSPHs was measured by size‐exclusion chromatographic and high‐performance liquid chromatography method (SEC–HPLC) on the basis of the previous method described by Noman et al. (2020). Initially, the analysis conditions were as follows: Shodex OHpak SB−803 HQ GPC column was used and the UV absorption of wavelength was set at 220 nm. Mobile phase composition was acetonitrile–water–trifluoroacetic acid (20:80:0.2, v/v/v), mobile phase flow rate was 0.5 ml/min. Column temperature was 30℃ and injection volume was 20 µl.

The molecular weight distribution of WJSPHs was calculated according to calibration curve of the following standards: RNase A (13,700 Da), human Insulin (5,808 Da), thymosin α_1_ (3,108 Da), somatostatin (1,638 Da), glycine–glycine–glycine (189 Da).

#### 2.4.4 Amino acid composition

The amino acid compositions of WJSP and WJSPHs were analyzed according to the previous method described by You et al. (2009). The amino acid composition of WJSP and WJSPHs were measured via an automatic amino acid analyzer L−8800 (Hitachi, Tokyo, Japan). Briefly, 30 mg of hydrolysates sample was mixed with 4 ml of 6 mol/L HCl solution for hydrolysis under a nitrogen atmosphere at 110℃ for 24 h. The amino acid contents were presented to 1 g amino acid per 100 g protein or hydrolysates.

#### 2.4.5 Circular dichroism (CD) spectroscopy

CD spectroscopy is a commonly used method to analyze the secondary structure changes. In this study, CD spectroscopy of WJSP and WJSPHs was scanned on the basis of the modified method of Zhang et al. (2020) on a JASCO J−1100 CD spectrometer (Jasco Corp, Tokyo, Japan) in the far–UV range of 180–260 nm. The measurement parameters were as follow: Scan speed was 100 nm/min, bandwidth was 1.0 nm, response time was 0.50 s, and step resolution was 1.0 nm at 25℃.

#### 2.4.6 Fourier transforms infrared (FTIR) spectroscopy

FTIR spectroscopy of WJSP and WJSPHs were determined on the basis of the method described by Zhang et al. (2020). Briefly, FTIR spectroscopy of WJSP and WJSPHs samples were obtained via a Fourier–transform infrared spectrometer (Nicolet 380, Thermo Scientific, UK) in the range of 4,000 and 400 cm^−1^ at 25 ℃. The resolution was 4 cm^−1^, and scans were subject to 32 times. Then, the spectra of each sample were smoothened, baseline corrected, normalized, and averaged for qualitative interpretation of spectra. Deconvolution of amide I was performed in the wavelength region of 1640−1710 cm^−1^ (Sow & Yang, 2015).

#### 2.4.7 Relative fluorescence intensity

The relative fluorescence intensity of WJSP and WJSPHs were measured based on the method of Yin et al. (2008) with minor modification. Initially, WJSP and WJSPHs were prepared in phosphate buffer solution (0.01 M, pH 7.0). Then, 20 µl of solution of 8‐anilino‐1‐naphthalenesulfonic acid (ANS, 8 mM) was mixed with 4 ml of sample solution, and the mixtures were shaken for 5 s. And the fluorescence intensity of samples was scanned in a spectrofluorometer (F−2700, Hitachi, Japan) in the wavelength region of 390–700 nm with the excitation wavelength of 375 nm. Both the excitation and emission slit widths were set at 5 nm.

#### 2.4.8 Thermal properties

The thermal stability of WJSP and WJSPHs was analyzed based on the method described by Shevkani et al. (2015). WJSP and WJSPHs samples were dissolved in distilled water to obtain a 15% sample solution (w/v) and stored at 4℃ for 24 h. The sample solution was accurately weighed and sealed hermetically into an aluminum crucible; then another new crucible was regarded as a blank reference. The thermal stability was scanned via the Mettler Toledo DSC 3 (Mettler Toledo, USA). The flow rate of nitrogen was sat at 50 L/min and the scanning temperature range was 25−180℃ with the heating rate of 5℃/min.

### 2.5 Determination of antioxidant ability of WJSP and WJSPHs

#### 2.5.1 DPPH radicals‐scavenging ability assay

The DPPH radicals‐scavenging ability of WJSP and WJSPHs were determined on the basis of the methods of Tang et al. (2009) with minor revisions. Initially, 1 ml of sample solution was mixed with 1 ml DPPH solution (4 mg in 100 ml 95% ethanol). Then the mixture was shaken and kept in the dark for 30 min; finally, the absorbance of each sample solution was determined by using the spectrophotometer (Shimadzu UV−1800, Tokyo, Japan) at 517 nm. Ascorbic acid (Vc) and GSH were used as the positive standard, and DPPH radicals scavenging ability was measured as follows:

$$S=\frac{A_{0}-A_{1}+A_{2}}{A_{0}}\;\times \;100\%$$

where *S* is the scavenging ability of DPPH radicals, *A*_0_ the absorbance of control, *A*_1_ the absorbance of each sample, *A*_2_ the absorbance of 0.5 ml of water and 0.5 ml of 95% ethanol.

#### 2.5.2 ABTS+ radicals‐scavenging ability assay

The ABTS^+^ radicals‐scavenging ability of WJSP and WJSPHs was analyzed based on the methods of Durak et al. (2013) with minor modifications. About 5 ml of ABTS solution (7.4 mmol/L) and 88 µl of potassium persulfate (2.6 mmol/L) were mixed and kept overnight in the dark at 25℃ for 12–16 h. Then, 0.4 ml of ABTS stock solution was diluted with phosphate buffer solution to keep the absorbance of 0.7 ± 0.02 at 734 nm. A volume of 10 µl of sample solution was added into 200 µl ABTS^+^ reaction solution and reacted at room temperature for 6 min in the dark. The absorbance of the sample solution was determined by using the microplate reader at 734 nm. Vc and GSH were used as the positive standard, and ABTS^+^ radical scavenging ability was measured as follows:

$$S=\frac{A_{0}-A_{1}}{A_{0}}\;\times \;100\%$$

where *S* represented scavenging ability of ABTS^+^ radicals, *A*_0_ the absorbance of phosphate buffer solution, and *A*_1_ the absorbance of each sample.

#### 2.5.3 Superoxide anion radicals‐scavenging ability assay

The superoxide anion radical (O_2_^–^)‐scavenging ability of WJSP and WJSPHs was determined based on the methods of Xie et al. (2016) with minor modifications. Initially, 0.5 ml of different concentrations of samples solution was added into 5 ml of Tris–HCl buffer solution (0.05 M, pH = 8.2) and reacted at 25℃ in a water bath for 20 min. Subsequently, the mixture was mixed with 0.5 ml of pyrogallol solution (7 mmol/L) preheated at 25℃. The absorbance of mixture was determined at 325 nm every 10 s for the total time of 5 min. Vc and GSH were used as the positive standards, and superoxide anion radicals‐scavenging ability was measured as follows:

$$S=\frac{A_{0}-A_{1}+A_{2}}{A_{0}}\;\times \;100\%$$

where *S* represents scavenging ability of superoxide anion radicals, *A*_0_ the absorbance of control, *A*_1_ the absorbance of each sample, and *A*_2_ the absorbance of distilled water replaced pyrogallol solution.

#### 2.5.4 Hydroxyl radicals‐scavenging ability assay

The hydroxyl radical (·OH)‐scavenging ability of WJSP and WJSPHs was measured as described by Xie et al. (2019) with slightly modification. Briefly, a 1.0 ml sample solution was added into the mixture including 1.0 ml of FeSO_4_ solution (2 mM) and 1.0 ml of salicylic acid–ethanol solution (6 mM). Subsequently, 1.0 ml of H_2_O_2_ (6 mM) was mixed with the resulting solution, and incubated in a 37℃ water bath for 30 min. Finally, the absorbance of obtained solution was determined at 510 nm. Vc and GSH were used as the positive standards, and hydroxyl radicals‐scavenging activity was measured as follows:

$$S=\frac{A_{1}-A_{0}}{A_{2}-A_{0}}\;\times \;100\%$$

where *S* represents the scavenging ability of hydroxyl radicals, *A*_2_ the absorbance of each sample, *A*_1_ the absorbance of distilled water replaced H_2_O_2_, and *A*_0_ the absorbance of distilled water replaced sample.

### 2.6 Determination of antioxidant activity of WJSP and WJSPHs in HepG2 cell

#### 2.6.1 Cell culture

Hcuman hepatocellular carcinomas cells (HepG2) were cultured in high‐glucose Dulbecco's modified Eagle's medium (DMEM) containing 25 mM glucose, 10% fetal bovine serum (FBS, v/v), 1% penicillin and streptomycin, and were incubated at 37℃ under a humidified atmosphere of 5% CO_2_.

#### 2.6.2 Cell viability assay

Cell viability of HepG2 was assessed by the CCK‐8 method. Briefly, HepG2 cells were seeded with the density of 1.0 × 10^5^ cells/ml onto 96‐well plates for 24 h under the condition of 5% CO_2_ and 90% relative humidity at 37℃. After 24 h incubation with different concentrations of WJSPHs, 10 µl CCK‐8 solution was added and reacted for 0.5−2 h and the absorbance of each well was determined at 450 nm.

#### 2.6.3 Determination of cell viability induced by H_2_O_2_ and its repair effect by WJSPHs

HepG2 cells were seeded with the density of 1.0 × 10^4^ cells/well in 96‐well plate for 24 h under the condition of 5% CO_2_ and 90% relative humidity at 37℃. Then cells were treated with complete DMEM containing different concentrations of H_2_O_2_ solution (50, 100, 150, 200, 250, 300, 400, 500, 600, 800, 1000, 2000 µM) to induce oxidative stress for 2 h under the conditions of 5% CO_2_ and 37℃. Cell viability was assessed by using the CCK‐8 method according to Section 2.6.2.

To appraise the effects of WJSPHs on the oxidative stress of HepG2 cells induced by H_2_O_2_, the cell viability was determined on the basis of the method described by Wen et al (2020). HepG2 cells were seeded with the density of 1.0 × 10^4^ cells/well in 96‐well plate for 24 h under the condition of 5% CO_2_ and 90% relative humidity at 37℃. Then cells were treated with 200 µM H_2_O_2_ for 2 h. Afterward, cells were washed with phosphate buffer and treated with WJSPHs solution at different concentrations (25, 50, 100, 200 µg/ml) for 24 h under the conditions of 5% CO_2_ and 37℃. Cell viability assays were performed by using the CCK‐8 method according to Section 2.6.2. HepG2 cells images were performed with Calcein–AM Staining Kit by ImageJ software.

#### 2.6.4 ROS level of WJSPHs on oxidative stress‐induced HepG2 cells

The production of ROS was measured via a fluorescent probe, which was 2′,7′‐dichlorofluorescein diacetates (DCFH–DA). HepG2 cells were seeded with the density of 1×10^5^ cells/ml in 6‐well plates. Both suspended and adherent cells were collected and cleansed with phosphate buffer, and incubated with DCFH–DA for 30 min at 37℃. After incubation, HepG2 cells were cleansed with phosphate buffer and dispersed in 200 µl phosphate buffer. Finally, the production of ROS was analyzed by using flow cytometry.

#### 2.6.5 SOD, GSH‐Px, and MDA production of WJSPHs in oxidative stress‐induced HepG2 cells

HepG2 cells were seeded with the density of 1×10^5^ cells/ml in 6‐well plates for 24 h with the condition of 5% CO_2_ and 90% relative humidity at 37℃, and then treated with 200 µM H_2_O_2_ for 2 h. Then, cells were washed with PBS and treated with different concentrations of WJSPHs solution (25, 50, 100, 200 µg/ml) for 24 h under the conditions of 5% CO_2_ and 37℃. The activity of SOD and GSH‐Px, and the production of MDA in HepG2 cell were measured by a biochemical analysis kit.

### 2.7 ACE inhibitory activity assay

The ACE inhibitory activity of WJSPHs was measured according to the methods described by Wu and Ding (2002) with slightly modification. Initially, 10 µl of sample solution in 100 mM borate buffer (pH 8.3, 300 mmol/L NaCl) was blended with 5 µl of ACE (100 mU/mL), and preincubated for 5 min at 37℃. Then the mixture was added into 50 µl of 6.5 mM hippuryl–histidyl–leucine solution (HHL, pH 8.3, dissolved in 100 mmol/L borate buffer containing 300 mmol/L NaCl) and incubated for 30 min at 37℃. About 85 µl of HCl (1 mol/L) was added to terminate the enzyme reaction. The reaction mixture was filtered through a 0.45 µm nylon syringe filter for HPLC analysis. Captopril was used as the positive control group.

The conditions of HPLC were as follows: Sepax Bio‐C18 column (4.6 × 250 mm, particle size 5 µm) was used and the UV absorption of wavelength was set at 228 nm. The mobile phase was acetonitrile–ultrapure water (15:85, both of them containing 0.05% TFA and 0.1% triethylamine). Then, the flow rate was 1 ml/min. The inhibitory activity of ACE was calculated as follow:

$$S=\frac{A_{0}-A}{A_{0}}\;\times \;100\%$$

where *A*_0_ is the chromatographic peak areas of borate buffer instead of sample and *A* the chromatographic peak areas of sample solution.

### 2.8 Statistical analysis

All experiments were performed at least three times. All data were presented as mean value ± standard deviation. Data analyses were conducted using SPSS 22.0 and Origin 2021 software. For multiple comparisons, all data were analyzed by One–Way ANOVA (Turkey's post hoc) and paired *t*‐test to determine statistical significance. *p* < 0.05 was defined as statistically significant difference between the groups.

## 3 RESULTS AND DISCUSSION

### 3.1 Preparation of WJSPHs

Protease plays a key role in the process of enzymatic hydrolysis. Different proteases usually show different enzyme sites, which can influence the degree of hydrolysis (DH), molecular weight distribution, and amino acid composition of hydrolysates, to further influence physico‐chemical properties and biological activities of hydrolysates (Betancur‐Ancona et al., 2014). In this study, DH and DPPH radical scavenging ability were used as reference index for the preparation of optimum protease. And then SDS‐PAGE electrophoresis and SEM were studied as reference indicators for the molecular weight distribution and structure of protein hydrolysates obtained from alcalase, neutrase, and papain.

**FIGURE 1:**
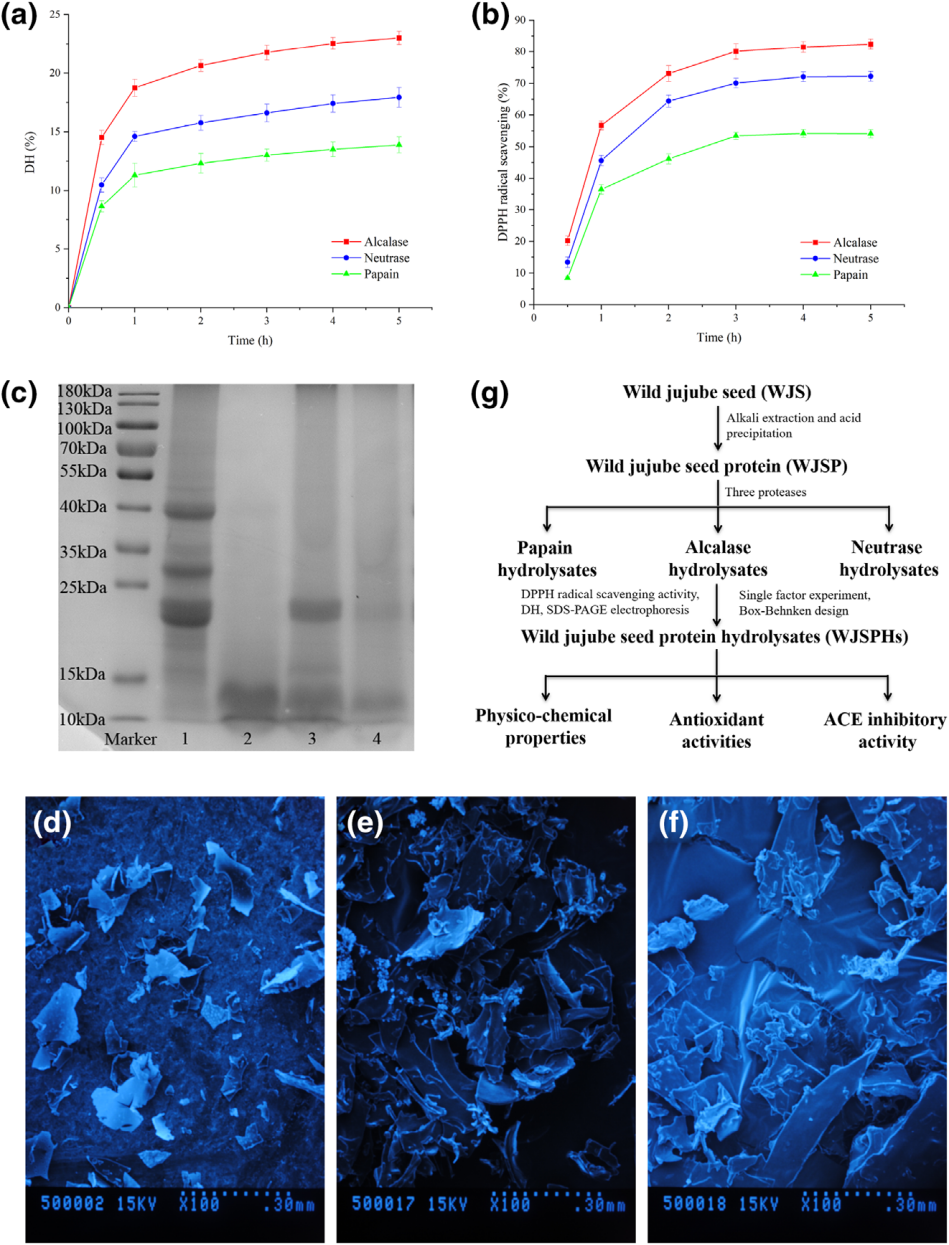
(a) DH, and (b) DPPH radical‐scavenging activity of WJSPHs obtained with alcalase, neutrase, and papain. (c) SDS‐PAGE electrophoresis of alcalase hydrolysates, neutrase hydrolysates, and papain hydrolysates. Line 1, WJSP; Line 2−4, alcalase hydrolysates, neutrase hydrolysates, and protamex hydrolysates. (d–f) SEM of papain hydrolysates, alcalase hydrolysates, and neutrase hydrolysates. (g) Preparation of WJSPHs

The DH curves of different hydrolysates of WJSP are shown in Figure . In the first hour, DH of different hydrolysates was significantly increased with the increment of reaction time. Then the growth rate of DH began to slow down after 2 h of reaction. At the end of reaction time, alcalase hydrolysates showed the best DH value of 23.01 ± 0.57%, followed by neutrase hydrolysates (17.94 ± 0.84%), and papain hydrolysates (13.89 ± 0.69%), respectively. In addition, DPPH radical‐scavenging activity of different hydrolysates is shown in Figure 1b; the order of free radical‐scavenging ability is alcalase hydrolysates (82.41 ± 1.60%), neutrase hydrolysates (72.23 ± 1.57%), and papain hydrolysates (54.31 ± 1.31%), respectively.

In addition, as depicted in Figure 1c, three kinds of hydrolysates all exhibited a band under 15 kDa in lines 2−4, and an obvious shift from high molecular weight bands (40–15 kDa) to lower molecular weight (< 15 kDa) was observed. In addition, neutrase hydrolysates and papain hydrolysates still showed some bands in the range of 25‒15 kDa. Furthermore, SEM of three hydrolysates indicated that alcalase hydrolysates showed more irregular structures and smaller pieces compared with neutrase hydrolysates and papain hydrolysates. The previously given results showed that alcalase can make the enzymolysis highly sufficient. Therefore, alcalase was selected as the best protease and used for followed studies due to its high DH value, antioxidant activity, and hydrolysis capacity (Figure 1g).

Based on the earlier‐mentioned results, the optimum preparation condition of WJSPHs was obtained by single factor experiment and Box–Behnken design: The substrate concentration was 5% (w/v), the enzyme:substrate ratio was 2%, the reaction time of enzymolysis was 3 h, and the pH of mixture was 8.6.

### 3.2 Physico‐chemical characteristics of WJSPHs

#### 3.2.1 SDS‐PAGE and Tricine‐SDS‐PAGE electrophoresis

SDS‐PAGE electrophoresis is commonly used for molecular weight and subunit analysis of protein and hydrolysates (Zhang et al., 2020). Tricine‐SDS‐PAGE can be used to isolate proteins and peptides with molecular weight of 1–10 kDa, and has become the main determination method for molecular weight of hydrolysates and polypeptides (Mundi & Aluko, 2012).

**FIGURE 2:**
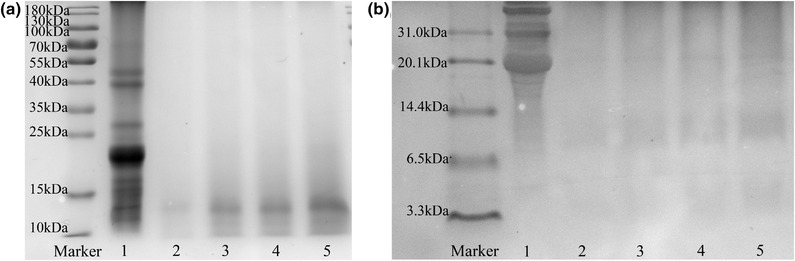
(a) SDS‐PAGE electrophoresis of WJSP and WJSPHs (alcalase hydrolysates of wild jujube seed protein). Line 1, WJSP; Line 2−5, different concentrations of WJSPHs (10, 20, 30, 40 mg/ml). (b) Tricine‐SDS‐PAGE electrophoresis of WJSP and WJSPHs. Line 1, WJSP; Line 2−6, different concentrations of WJSPHs (10, 20, 30, 40 mg/ml)

SDS‐PAGE and Tricine‐SDS‐PAGE images of WJSP and WJSPHs are shown in Figure 2. WJSP was mainly distributed in the wide molecular weight range in line 1, and its protein bands were mainly distributed in the range of 55–10 kDa. However, as shown in lines 2‒5, the 49, 40, 28, 23, and 20 kDa bands of WJSP had clearly disappeared after enzymatic hydrolysis. Low molecular weights under 15 kDa were present (Figure 2a). Furthermore, as shown in Figure 2b, Tricine–SDS–PAGE images indicate that WJSPHs exhibited few bands in the range of 14.4−6.5 kDa and 6.5−3.3 kDa, respectively, in lines 2‒6. These results indicate that WJSP was converted into a smaller molecular weight peptide after enzymatic hydrolysis by alcalase. This finding was similar to the previously reported one about black bean protein hydrolysates (Zheng et al., 2019), tree peony seed protein hydrolysates (Wang et al., 2021), and perilla seed meal protein hydrolysates (Kim & Yoon, 2020). Recent studies have shown that low molecular weight peptides exhibited better biological activities and were beneficial to be absorbed and utilized by the human body (Zheng et al., 2018). Therefore, the previously mentioned results suggest that WJSPHs may have high potential nutritive values with low molecular weight distribution.

#### 3.2.2 Molecular weight distribution of WJSPHs

**FIGURE 3:**
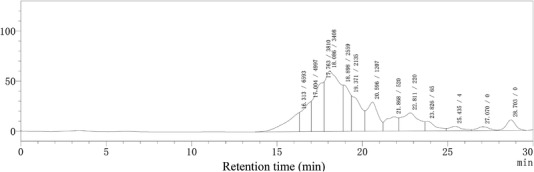
Size‐exclusion chromatographic separation determination of WJSPHs

The molecular weight distribution of WJSPHs is presented in Figure 3 and Table 1. Size‐exclusion chromatographic determination results indicated that WJSPHs contained polypeptides with various molecular weights. The molecular weight distribution of WJSPHs was mainly under 5,000 Da and accounted for 86.03% of the total hydrolysates, and the fraction with molecular weights under 1,000 Da was 17.76% of the total hydrolysates. Xie et al. (2019) reported that different molecular fractions from mung bean protein hydrolysates (MBPHs) exhibited different antioxidant ability and ACE inhibitory activity, where MBPHs–I (<3,000 Da) exhibited the strongest DPPH‐ and hydroxyl radicals‐scavenging activity. Zhang et al. (2019) also found that low molecular fractions (<1,000 Da) from protein hydrolysate of corn germ meal showed higher DPPH‐ and ABTS^+^ radicals‐scavenging activity than other fractions (1,000−3,000 Da and 3,000−10,000 Da). Previous reports indicated that low molecular weight fractions from protein hydrolysates exhibited stronger antioxidant ability than high molecular weight protein and hydrolysates (Zheng et al., 2018). Therefore, the results of this study show that enzymatic hydrolysis decreased the molecular weight of WJSP and achieved some low molecular weight peptides, which may possess better biological activities and can be used as potential peptides resources for the food industry.

**TABLE 1:** Molecular weight distribution of WJSPHs

MW (Da)	>10,000	10,000–5000	5,000–3,000	3,000–1,000	1,000–200	<200
Content (%)	–	13.97	37.52	26.75	13.50	4.26

#### 3.2.3 Amino acid composition

The amino acid composition of WJSP and WJSPHs are shown in Table 2. WJSPHs are rich in Asp (12.19 ± 0.15%), Glu (27.17 ± 0.32%), and Arg (10.52 ± 0.21%). Particularly, the high contents of Asp and Glu in WJSPHs were negatively charged amino acids (NCAA), which accounted for 39.36 ± 0.42% of the total amino acid content. The high content of NCAA is consistent with the previous reports wherein NCAA is the main amino acids of protein in some seed plants (Gao et al., 2018). Previous studies reported that NCAA exhibited strong antioxidant activity by providing electrons to scavenging free radicals (Girgih et al., 2015). Meanwhile, the content of aromatic amino acids (AAAs) in WJSPHs was 7.48 ± 0.06%, which was higher than that of WJSP (7.07 ± 0.04%, *p* < 0.05). It was reported that AAA can scavenge free radicals by providing protons to free radicals (Hou et al., 2017). Thus, the earlier‐mentioned results indicated that WJSPHs may exhibit antioxidant activity by converting free radicals to stable molecules. The hydrophobic amino acids (HAAs) in WJSPHs, such as Ala, Val, Leu, Ile, and Phe, comprised 25.12 ± 0.27% of the total amino acid content.

**TABLE 2:** Amino acid composition of WJSPHs and WJSP

Coding	Amino acid	WJSP (g/100 g)	WJSPHs (g/100 g)
1	Aspartic (Asp)	11.67 ± 0.13	12.19 ± 0.15
2	Threonine (Thr)	2.77 ± 0.09	2.87 ± 0.02
3	Serine (Ser)	5.47 ± 0.12	4.79 ± 0.05
4	Glutamic acid (Glu)	25.83 ± 0.31	27.17 ± 0.32
5	Glycine (Gly)	5.03 ± 0.07	5.42 ± 0.05
6	Alanine (Ala)	3.93 ± 0.02	4.04 ± 0.04
7	Cysteine (Cys)	0.83 ± 0.02	0.77 ± 0.01
8	Valine (Val)	5.81 ± 0.11	6.22 ± 0.03
9	Methionine (Met)	0.59 ± 0.05	0.08 ± 0.01
10	Isoleucine (Ile)	3.25 ± 0.03	3.11 ± 0.11
11	Leucine (Leu)	7.86 ± 0.16	7.46 ± 0.16
12	Tyrosine (Tyr)	2.87 ± 0.02	3.28 ± 0.25
13	Phenylalanine (Phe)	4.20 ± 0.13	4.20 ± 0.13
14	Lysine (Lys)	2.15 ± 0.02	4.04 ± 0.21
15	Histidine (His)	2.78 ± 0.06	2.90 ± 0.17
16	Arginine (Arg)	9.88 ± 0.30	10.52 ± 0.21
17	HAA^a^	25.64 ± 0.21	25.12 ± 0.27*
18	AAA^b^	7.07 ± 0.04	7.48 ± 0.06*
19	PCAA^c^	15.81 ± 0.33	16.46 ± 0.51*
20	NCAA^d^	37.5 ± 0.36	39.36 ± 0.42**
21	EAA^e^	26.63 ± 0.24	27.99 ± 0.37**

^a^ Hydrophobic amino acid.

^b^ Aromatic amino acids.

^c^ Positively charged amino acids.

^d^ Negatively charged amino acids.

^e^ Essential amino acid. Data values are expressed as the means ± standard deviation (*n* = 3),.

* *p *< 0.05 and

** *p *< 0.01 compared with WJSP.

Additionally, the essential amino acids (EAAs) content of WJSPHs, such as Thr, Val, Leu, Ile, Phe, and Lys, accounted for 27.99 ± 0.37% of the total amino acid content, and was higher than WJSP (26.63%, *p* < 0.01), suggesting that enzymatic hydrolysis could improve the nutritive value of WJSO. The EAA contents of WJSPHs reached the daily EAA content recommendation by WHO/FAO. These results suggest that WJSPHs exhibits an ideal amino acid composition and nutritional value.

#### 3.2.4 CD spectrum

**FIGURE 4:**
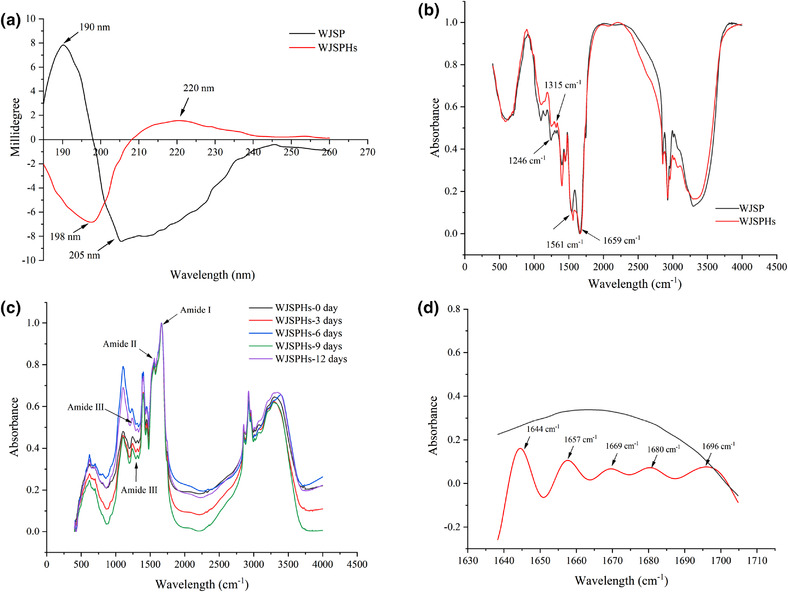
(a) CD spectrum of WJSP and WJSPHs, (b) FTIR spectrum of WJSP and WJSPHs, (c) FTIR average spectrum of WJSPHs, (d) Deconvolution of amide I of WJSPHs

The CD of WJSP and WJSPHs in the range of 190–260 nm is exhibited in Figure 4a. The CD spectrum of WJSP showed a strong positive band at 190 nm and a negative band at 205 nm, which are the characteristic absorption peaks of a–helix structure. However, CD spectrum of WJSPHs showed a strong negative peak at wavelength of 198 nm, and a weak and wide positive peak at wavelength of 220 nm, which indicates the presence of random coil structure. These results indicate that the secondary structure of WJSPHs changed from a–helix to random coil after enzymatic hydrolysis. It is worth noting that the previous report had also proof that black bean protein hydrolysates follow a similar transformation from a–helix structure to the random coil structure (Evangelho et al., 2017).

#### 3.2.5 FTIR spectrum

Fourier infrared absorption spectroscopy (FTIR) is a type of molecular absorption spectrum reflecting the vibrational energy level transitions of reactive molecules. It was used to analyze the secondary structure of proteins or hydrolysates according to the absorption wavelengths and intensities (Carbonaro & Nucara, 2010). The characteristic absorption band of protein mainly consists of three parts: Amide I band (1,600−1,700 cm^−1^), in which the absorption peaks of 1,646−1,661 cm^−1^ indicate a–helix structure, 1,610−1,640 cm^−1^ indicate β–sheets structure, and 1,660−1,695 cm^−1^ indicate β–turn structure, respectively. Amide III band (1,220−1,330 cm^−1^): The absorption peaks at 1,290−1,330 cm^−1^ indicate a–helix structure, the absorption peaks at 1,220−1,250 cm^−1^ indicate a β–sheets structure, and the absorption peaks at 1,265−1,295 cm^−1^ indicate a β–turn structure, and the absorption peaks of 1,245−1,270 cm^−1^ represent the random coil, respectively. Amide II band (1,500−1,600 cm^−1^): Represents the structure of N–H.

Three characteristic absorption bands can be distinctly observed according to Figure 4b, amide I: 1,659 cm^−1^ absorption peak indicates an a–helix structure. Amide III band: 1,246 cm^−1^ absorption peak indicates random coil structure; 1,315 cm^−1^ absorption peak indicates an a–helix structure. Amide Ⅱ band: 1,561 cm^−1^ absorption peak indicates N–H bending structure. Then, we analyzed the structural stability of WJSPHs during 12 days by using average spectrum (Figure 4c and Table 3). The peak location of amide Ⅰ, amide Ⅱ, and amide III was relatively stable and not significantly changed after 12 days. Furthermore, we further studied the deconvolution of amide I in the wavelength range of 1640−1710 cm^−1^. As shown in Figure 4d, there were 5 absorption peaks in amide Ⅰ: 1644 cm^−1^ and 1657 cm^−1^ absorption peaks were the major peaks and indicated a–helix structure, and 1669 cm^−1^, 1680 cm^−1^, and 1696 cm^−1^ indicated β–turn structure. These results suggest that secondary structure of WJSPHs was stable, which mainly included random coil structure and still contained a–helix and β–turn structure after enzymatic hydrolysis. Combined with the results of CD spectrum, this indicates that the secondary structure of WJSPHs was mainly random coil with loose structure, which may increase its solubility, and may be beneficial for the digestion and absorption by the human body. Similarly, Xie et al (2019) also found unopened the a–helix and β–turn structure in the secondary structure of mung bean protein hydrolysates.

**TABLE 3:** Assignment of the peaks identified in FTIR average spectra

Region	Wavelength (cm^‐1^)	Assignment
Amide I	1659 ± 1	a–helix structure
Amide II	1560 ± 2	N–H bend
Amide III	1313 ± 3	a–helix structure
	1247 ± 2	random coil structure

#### 3.2.6 Relative fluorescence intensity

**FIGURE 5:**
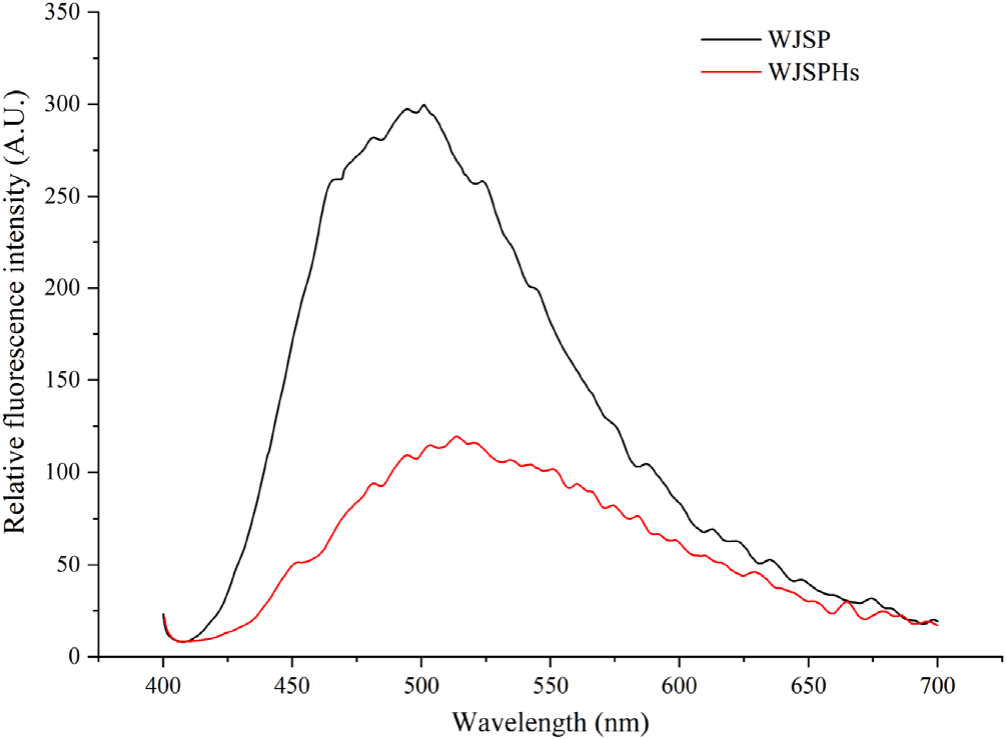
Relative fluorescence intensity of WJSP and WJSPHs

8‐Aniline‐1 naphthalene sulfonic acid (ANS) is a common fluorescent dye, which tends to show a higher affinity to the hydrophobic region of unfolded proteins and hydrolysates, which shows a positive correlation between hydrophobicity and relative fluorescence intensity (Pallarès et al., 2004). As shown in Figure 5, the relative fluorescence intensity of WJSPHs reached the maximum value of 119.52 at 510 nm, which was much lower than maximum fluorescence intensity of WJSP (300.51). This may be attributed to the re‐burying of hydrophobic amino acids and exposure of hydrophilic groups, thus further reducing its relative fluorescence intensity (Zheng et al., 2019). This result is similar to the previous hydrolysates report, such as the ones for black bean and tree peony seed protein hydrolysates, suggesting that protease hydrolysis reduces the relative fluorescence intensity of WJSP, and further exposes hydrophilic groups and enhances its solubility.

#### 3.2.7 Thermal properties

**FIGURE 6:**
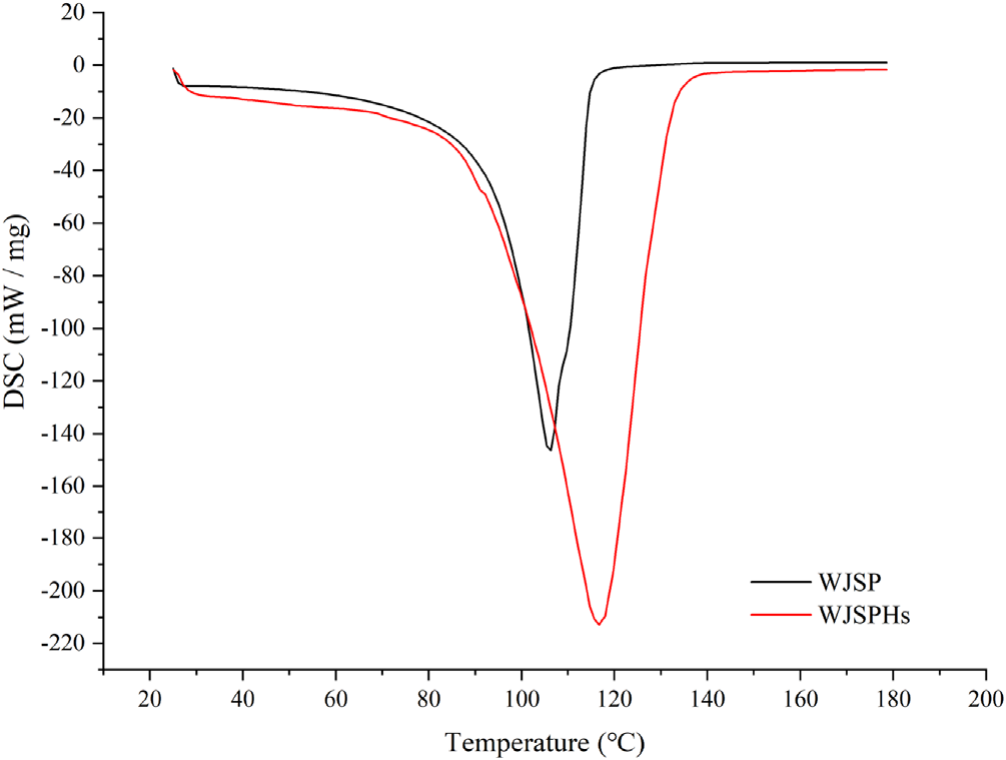
DSC profiles of WJSP and WJSPHs

Differential scanning calorimetry (DSC) is the most widely used thermal analysis technology, which can reflect the thermal properties of protein. Denaturation temperature (*T*_d_) can effectively reflect the thermal stability of protein and hydrolysates: The higher the *T*_d_ value, the higher the thermal stability. The *∆H* is the energy required for denaturation, which can reflect the structure stability during the heating process: the higher the *∆H* value, the more stable the structure. As shown in Figure 6, with the increase of temperature, the structures of WJSP and WJSPHs became unstable. In addition, the highest thermal denaturation temperature of WJSPH was 119.29℃, which was higher than that for WJSP (110.5℃) as well as for other common seed protein resources given in previous reports, such as *Amygdalus pedunculata* Pall seeds (91.58℃, Li et al., 2020) and hemp protein isolate (95℃, Wang et al., 2007). These results show that WJSPHs exhibit a higher thermal stability after enzymatic hydrolysis. Great thermal stability is conducive to the better application of WJSPHs and may improve its application value in functional food processing.

### 3.3 Determination of the antioxidant activity

#### 3.3.1 DPPH radicals‐scavenging activity

As a stable radical in alcohol solution, DPPH has the maximum absorbance wavelength at 517 nm. When a hydrogen donor such as antioxidant was encountered, the single electron of the radical combined with hydrogen, and the absorbance intensity was decreased at 517 nm. The degree of reduction of absorbance has a quantitative relationship with the degree of free radical removal. Thus, DPPH radicals have been widely regarded as an indicator to evaluate the scavenging ability of antioxidants (Ghribr et al., 2015).

**FIGURE 7:**
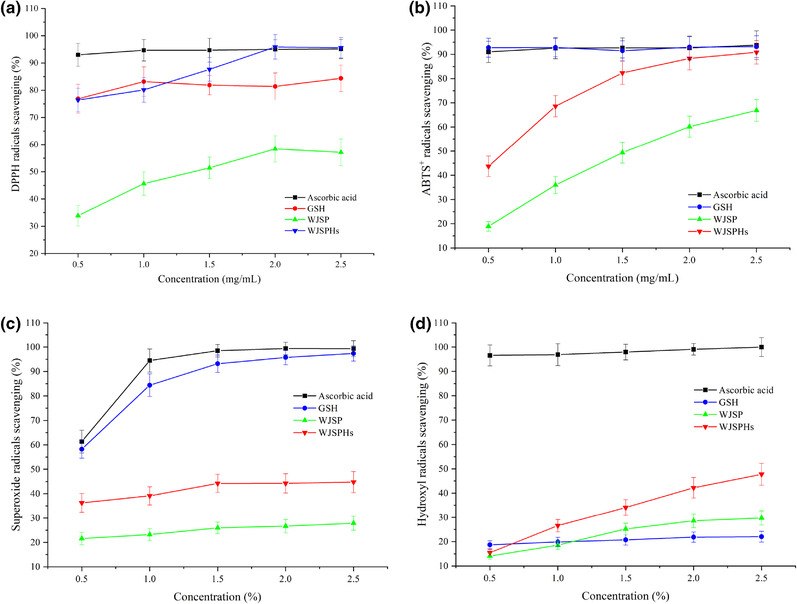
In vitro antioxidant ability of WJSPHs and WJSP. (a) DPPH radicals‐scavenging ability; (b) ABTS^+^ radicals‐scavenging ability; (c) superoxide radicals‐scavenging ability; (d) hydroxyl radicals‐scavenging ability. Results are the mean values of triplicate determinations on the sample ± standard deviations

The DPPH radicals‐scavenging activities of WJSP and WJSPHs are shown in Figure 7a. Within the concentration range of 0.5−2.5 mg/ml, the scavenging activities of all samples were dose‐dependent; they exhibited higher scavenging capability with increase in concentration until the radicals were wiped out. It was reported that hydrolysis was beneficial to improve the DPPH radicals‐scavenging capability. In the present study, WJSPHs exhibited higher DPPH radicals‐scavenging activity in comparison with WJSP (*p* < 0.05) under the same concentration. In the range of 1.5−2.5 mg/ml, WJSPHs exhibited stronger DPPH scavenging capability than GSH, which is a common antioxidant peptide and widely used in functional foods. At 2.5 mg/ml concentration, the DPPH radicals‐scavenging activity of WJSPHs achieved the maximum of 94.60% and was close to the maximum of ascorbic acid (96.03%). The given results indicate that WJSPHs exhibit stronger DPPH radicals‐scavenging activity than the WJSP.

#### 3.3.2 ABTS+ radicals‐scavenging activity

The ABTS^+^ radical‐scavenging activities of WJSP and WJSPHs at different concentrations are shown in Figure 7b. WJSP and WJSPHs showed high ABTS^+^ radical‐scavenging capability with the increase in the concentration in the range of 0.5−2.5 mg/ml. WJSPHs showed better ABTS^+^ radical‐scavenging capability than WJSPHs in the range of 0.5−2.5 mg/ml. Both WJSP and WJSPHs showed the highest scavenging ability at the 2.5 mg/ml concentration of 66.88% and 90.84%, respectively. The improvement in the scavenging activity of WJSPHs might be attributed to the increase of NCAA and AAA after enzymatic hydrolysis, which enhances the provision of protons to unpaired free radicals and thus, further enhances the free radical‐scavenging ability. It is worth noting that at 2.5 mg/ml concentration, WJSPHs exhibited the best ABTS^+^ radicals‐scavenging activity of 90.84%, which was close to the maximum of ascorbic acid (92.77%) and GSH (93.13%).

#### 3.3.3 Superoxide anion radicals (O_2_)‐scavenging activity

Superoxide anion radicals (O_2_) can produce hydrogen peroxide and hydroxyl radicals (·OH) through disproportionation. These free radicals and products will inhibit the activity of some enzymes in vivo and destroy the structure of cell membrane and DNA, resulting in the damage of the function of normal cells. Thus, the superoxide anion radicals‐scavenging activity can be used as an indicator to investigate the scavenging activity of antioxidants (Udenigwe et al., 2009).

The superoxide anion radicals‐scavenging activity of WJSP and WJSPHs is presented in Figure 7c. The scavenging activity of WJSP and WJSPHs are observed to be in a dose‐dependent manner and showed high scavenging ability with increase of concentration. Ascorbic acid exhibited the highest scavenging ability, followed by GSH and WJSPHs, whereas WJSP exhibited the lowest scavenging ability. WJSP and WJSPHs showed the best scavenging activity of 44.77% at 2.5 mg/ml concentration, which is higher than WJSP (27.86%). This result indicates that the enzymatic hydrolysis of WJSP is beneficial to improve its O_2_^–^ scavenging ability.

#### 3.3.4 Hydroxyl radicals (·OH)‐scavenging activity

>Hydroxyl radicals (·OH) are extremely active free radicals that are prone to chemical reaction. Hydroxyl radicals are favorable to react with some amino acids, proteins, DNA, and other biological molecules, which can cause lipid peroxidation. Hence, the scavenging activity of hydroxyl radicals can be used as an important index to evaluate antioxidant activity (Xie et al., 2008).

As shown in Figure 7d, hydroxyl radicals‐scavenging activity of WJSP and WJSPHs presented a dose‐dependent manner until the radicals were wiped out. Hydrolysates obtained from alcalase digestion exhibited higher hydroxyl radicals‐scavenging activity (*p* < 0.05) than WJSP over the range of concentration studied. In addition, WJSPHs reached the highest hydroxyl radicals‐scavenging activity at the 2.5 mg/ml concentration of 47.77%, which was much higher than the hydroxyl radicals‐scavenging activity of WJSP of 29.76%.

Therefore, these results demonstrate that enzymatic hydrolysis treatment can improve the free radicals‐scavenging capacity and show higher antioxidant activity than nonenzymatic proteins. These studies also illustrate that WJSPHs can be used as an antioxidant additive in functional foods.

### 3.4 Antioxidant activity of HepG2 cells

#### 3.4.1 Effect of WJSPHs on cell viability of HepG2 cells

**FIGURE 8:**
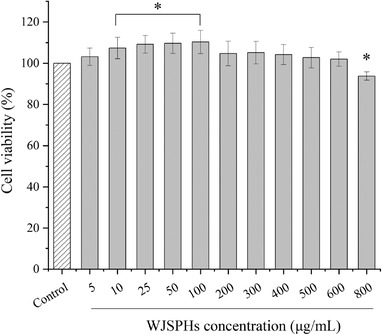
The cell viability of HepG2 cells treated with WJSPHs

As shown in Figure 8, HepG2 cells treated with 200, 300, 400, 500, and 600 µg/ml of WJSPHs showed no significant influence on cell viability (*p *> 0.05) compared with the control group. Moreover, the cell viability of HepG2 cells treated with 10, 25, 50, and 100 µg/ml of WJSPHs was improved (*p *> 0.05). These results demonstrate that WJSPHs show almost no cytotoxicity on HepG2 cells.

#### 3.4.2 Effect of WJSPHs on cell viability of HepG2 cells induced by H_2_O_2_

**FIGURE 9:**
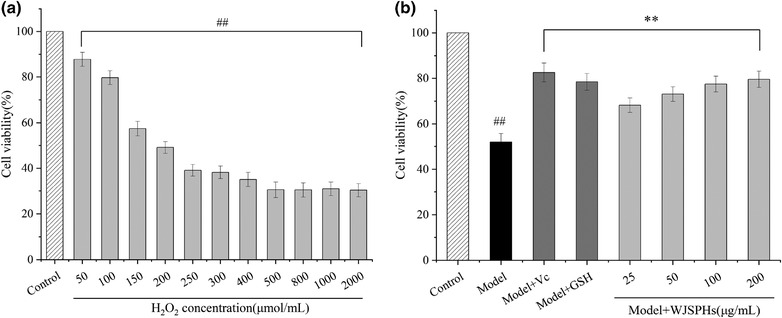
(a) The cell viability of HepG2 cells treated with H_2_O_2_, (b) Effect of WJSPHs on cell viability of HepG2 cell induced by H_2_O_2_. Data values are expressed as the means ± standard deviation (*n* = 3), ^#^ *p *< 0.05 and ^##^ *p *< 0.01 compared with the control group, ^*^ *p *< 0.05 and ^**^ *p *< 0.01 compared with the H_2_O_2_ group (Model)

In this study, the different concentrations of H_2_O_2_ (50, 100, 200, 400, 600, 800, 1000, 2000 µM) were used to induce HepG2 cells for 2 h to establish the oxidative stress model. As shown in Figure 9a, H_2_O_2_ decreased the viability of HepG2 cells in a concentration‐dependent manner. The cell viability of HepG2 was reduced to 62.08% by treating with 200 µM H_2_O_2_ for 2 h (*p *< 0.05 and *p *< 0.01) compared with the control group, while the oxidative stress was obviously observed and the cell viability was proper. Therefore, the concentration of 200 µM of H_2_O_2_ was used to induce the oxidative stress for all subsequent experiments.

Based on the oxidative stress model of HepG2 cells induced by H_2_O_2_, the effect of WJSPHs on oxidative stress cell was determined. As shown in Figure 9b, when cells were treated with 200 µM H_2_O_2_, the cell viability was significantly decreased, indicating HepG2 cell oxidative damage. Compared with the H_2_O_2_ group, WJSPHs increased the viability of HepG2 cells induced by 200 µM H_2_O_2_ in a dose‐dependent manner (*p* < 0.05 and *p* < 0.01), and showed the maximum cell viability value of 81.53%. The mentioned results indicate that WJSPHs can significantly decrease the injury due to oxidative stress of HepG2 cells induced by H_2_O_2_.

#### 3.4.3 Effect of WJSPHs on morphology of HepG2 cells induced by H_2_O_2_

The morphology of HepG2 cell of all groups is shown in Figure 11. Compared with the control group, the number of HepG2 cells in the model group was obviously decreased and the edge was not clear, which indicated that the H_2_O_2_–injured model was established successfully. After treatment with different concentrations of WJSPHs, the number of HepG2 cells was apparently increased and cell growth tends to be normal in a concentration‐dependent manner. The morphology of HepG2 cells of 200 µg/ml of WJSPHs group was close to the positive group (Vc group and GSH group). These results show that WJSPH can repair the oxidative damage of H_2_O_2_‐injured HepG2 cells.

#### 3.4.4 Effect of WJSPHs on ROS level of HepG2 cells induced by H_2_O_2_

**FIGURE 10:**
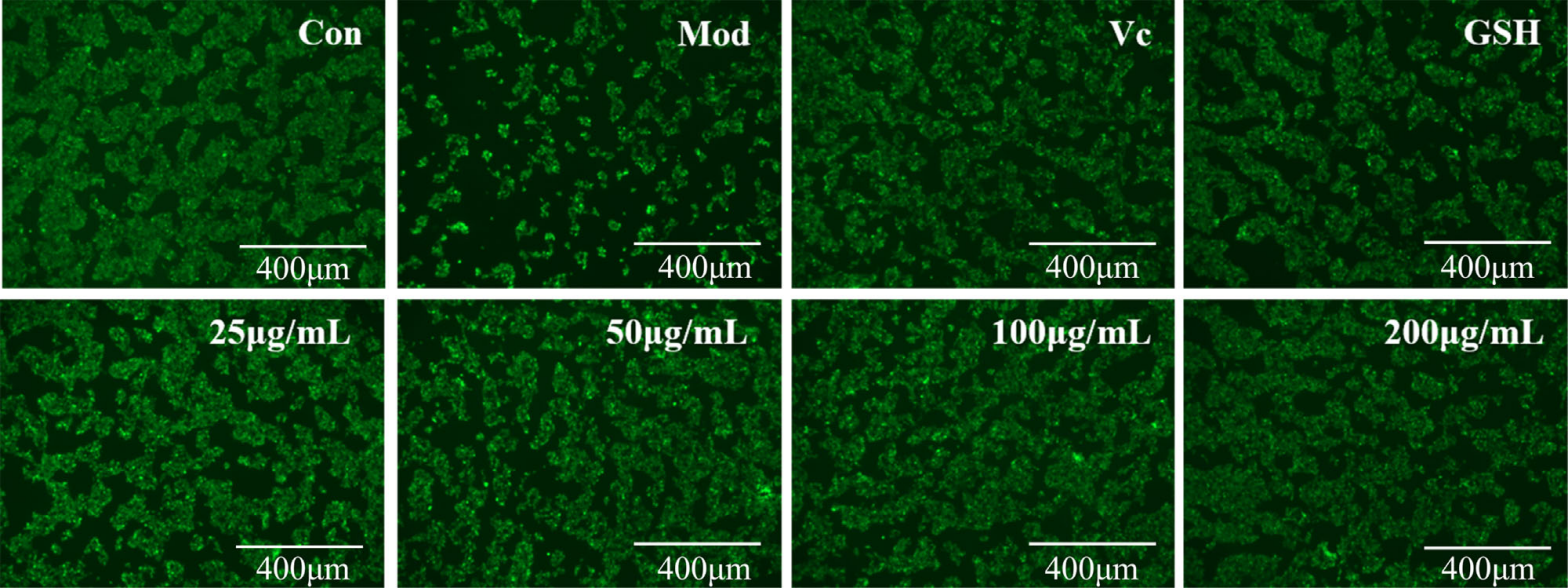
Effects of different concentrations of WJSPHs on ROS level of H_2_O_2_‐injured HepG2 cells. Data values are expressed as the means ± standard deviation (*n* = 3), ^#^ *p *< 0.05 and ^##^ *p *< 0.01 compared with the control group, ^*^ *p *< 0.05 and ^**^ *p *< 0.01 compared with the H_2_O_2_ group (Model)

As the intermediate products of normal metabolism of cells, reactive oxygen species (ROS), including singlet oxygen (^1^O_2_), superoxide radicals (O_2_^–^), hydrogen peroxide (H_2_O_2_), and hydroxyl radicals (·OH) play a significant role during normal human physiological and pathological processes (Hu et al., 2022). During some abnormal physiological processes, the over‐production of ROS may result in the oxidative damages of protein, DNA breaks, and the lipid peroxidation, which further lead to injury of the biomembrane and emergence of some chronic diseases (Zhu et al., 2021). Therefore, the production of ROS can be used as a key indicator of oxidative stress. This study investigated the influence of WJSPHs on ROS production in H_2_O_2_‐injured HepG2 cells. As described in Figure 10a, compared with the control group, H_2_O_2_ increased the release of mitochondrial ROS of HepG2 cells (*p* < 0.01). After treatment with different concentrations of WJSPHs (25, 50, 100, 200 µg/ml) for 24 h, the production of mitochondrial ROS was significantly reduced when compared with the model group. The production of ROS at 100 and 200 µg/ml of WJSPHs was closed to the positive group of Vc and GSH. These results demonstrate that WJSPHs can inhibit the generation of ROS and reduce the oxidative stress in HepG2 cells. Yi et al (2020) also reported that peptides from soybean protein hydrolysates inhibited the ROS level generated by H_2_O_2_. Therefore, WJSPHs may play a positive protective effect on oxidative stress damage.

**FIGURE 11:**
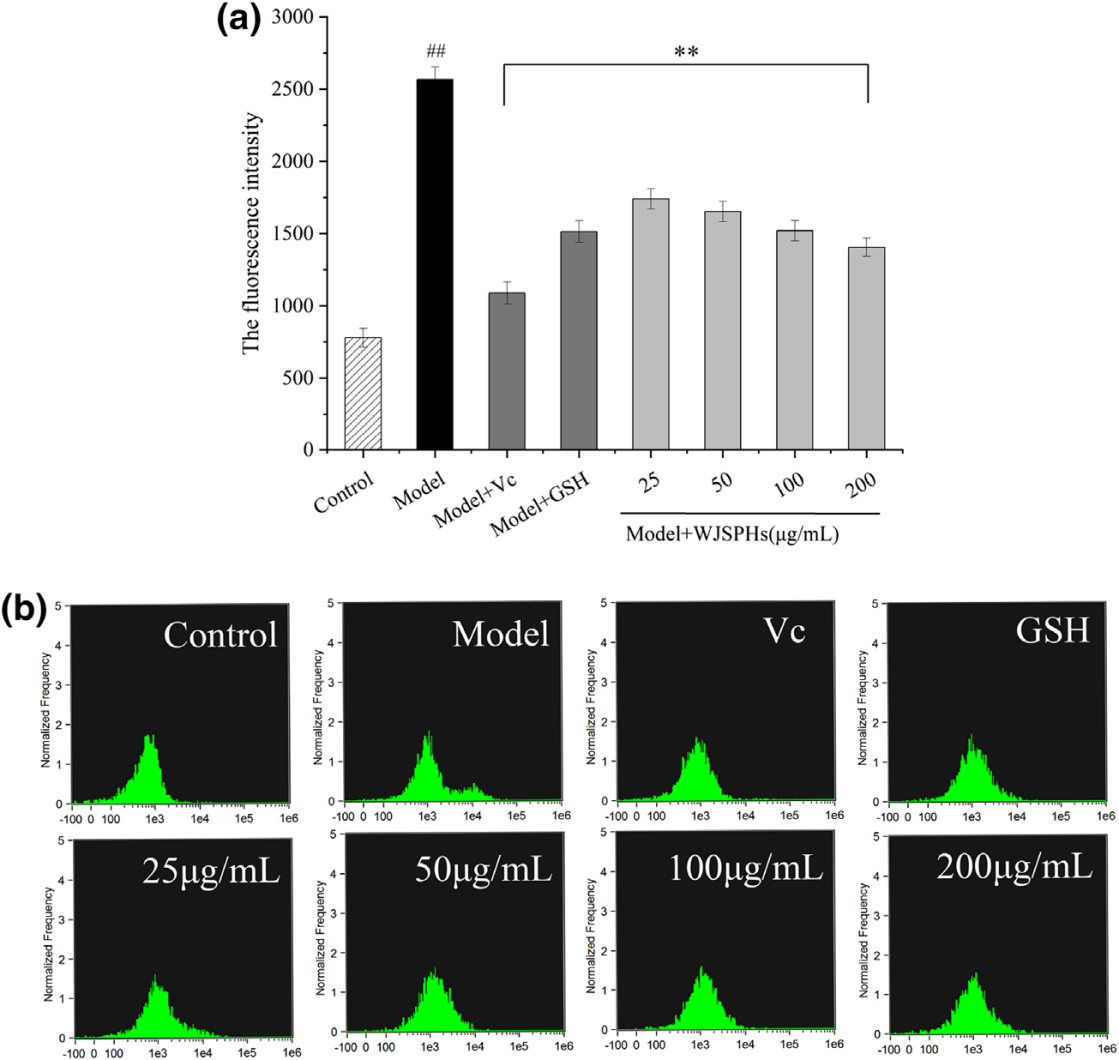
Effects of ascorbic acid (Vc), GSH, and different concentrations of WJSPHs (25, 50, 100, 200 µg/ml) on the calcein–AM image of injured HepG2 cells

#### 3.4.5 Effect of WJSPHs on MDA level and activity of SOD and GSH–Px of HepG2 cells induced by H_2_O_2_

**FIGURE 12:**
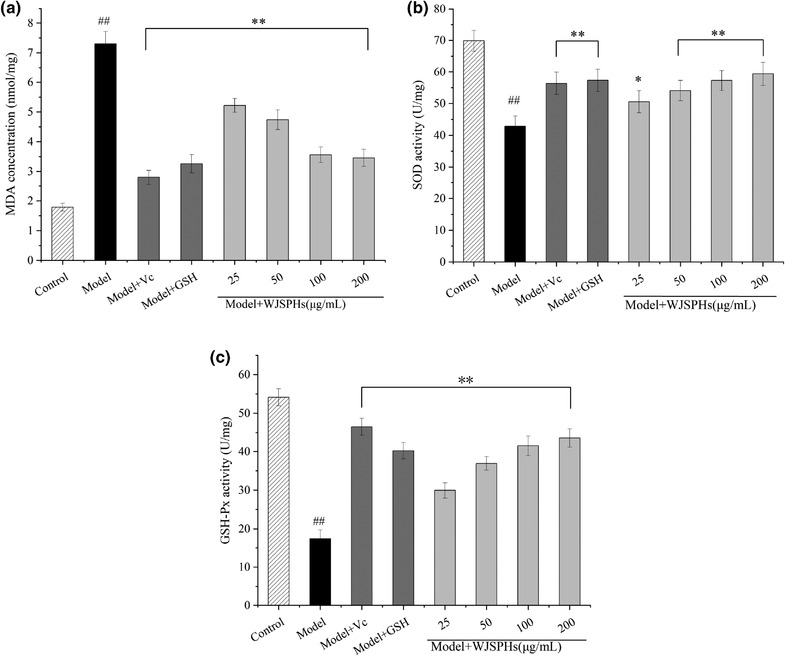
Effects of different concentrations of WJSPHs on (a) MDA level, (b) SOD activity, and (c) GSH‐Px of H_2_O_2_–injured HepG2 cells. Data values are expressed as the means ± standard deviation (*n* = 3), ^#^ *p *< 0.05 and ^##^ *p *< 0.01 compared with the control group, ^*^ *p *< 0.05 and ^**^ *p* < 0.01 compared with the H_2_O_2_ group (Model)

Our previous studies had shown that WJSPHs exhibited promising free radical‐scavenging ability in vitro, which may alleviate the oxidative stress damage in HepG2 cells. According to the previous reports, excessive free radicals could induce lipid peroxidation, and MDA is the degradation product of lipid peroxidation, which can indirectly reflect the content of free radicals in tissues (Fukuda et al., 2007). Figure 12a shows the effect of WJSPHs on the MDA level; the model group treated with H_2_O_2_ increased the release of MDA when compared with the control group, which indicates that free radicals induced by H_2_O_2_ caused occurrence of lipid oxidation. The Vc group and GSH group can apparently prevent the release of MDA and was gradually closed to the control group. In addition, the levels of MDA of the WJSPHs groups were significantly reduced in a dose‐dependent manner compared with the H_2_O_2_ group (*p *< 0.05). These results indicate that WJSPHs show good antioxidant activity via free radical‐scavenging mechanism, to further reduce the lipid oxidation caused by excessive free radicals.

Superoxide dismutase is an important endogenous antioxidant enzyme, which can catalyze the transformation of superoxide anion radical, and thus can play an antioxidant role by scavenging the harmful superoxide radical (Yi et al., 2020). The GSH‐Px is an important peroxidase widely existing in the body, which can promote the reduction of toxic and harmful peroxides to non‐toxic hydroxyl compounds so as to further protect cells and the body from peroxide damage (Liang et al., 2017). However, excessive free radicals can also break the antioxidant gene and decrease the activity of some antioxidant enzymes, resulting in intracellular oxidative damage. Therefore, the activities of antioxidant enzymes, such as SOD and GSH‐Px, are regarded as a vital indicator of antioxidant ability. As shown in Figure 12b,c, the activities of SOD and GSH‐Px of H_2_O_2_ group were obviously decreased when compared to the control group (*p* < 0.05), which indicates that H_2_O_2_ treatment induced significant oxidative injury to HepG2 cells. By comparison, WJSPHs treatment obviously improved the activity of SOD and GSH‐Px of HepG2 cells induced by H_2_O_2_ (*p* < 0.05) in a dose‐dependent manner. The activities of SOD and GSH–Px at 100 and 200 µg/ml of WJSPHs group were close to the positive group (Vc and GSH group). These results suggest that WJSPHs can protect the antioxidant enzyme activity and remove the free radicals to further prevent the oxidative damage induced by H_2_O_2_. Similarly, Wang et al (2021) reported that antioxidant peptides from Antarctic Krill hydrolysates showed high scavenging activities on DPPH, ·OH, O_2_^–^ free radicals, and simultaneously scavenged excess ROS, increased the activity of antioxidant enzymes, and decreased the MDA content.

Consequently, WJSPHs show a promising protective effect on oxidative stress damage by scavenging intracellular free radicals, improving the activity of antioxidant enzymes, and inhibiting lipid oxidation in HepG2 cells. Our findings suggest that WJSPHs may be a promising antioxidant to prevent oxidative‐related diseases in the health food industry.

### 3.5 ACE inhibitory activity of WJSPHs

Hypertension is the most common chronic disease and the main risk factor of cardiovascular and cerebrovascular diseases. ACE is a key factor to promote the conversion of angiotensin II, which can further lead to hypertension. Therefore, the inhibitor of ACE was used as an effective drug to treat hypertension. At present, drugs for the treatment of hypertension are mainly β‐receptor blockers, calcium channel blockers, diuretics, and ACE inhibitors. However, regular use of these synthetic drugs lead to stomach damage (Kheeree et al., 2020). Previous reports had shown that peptides obtained from food protein hydrolysates exhibited high inhibitory activity of ACE, even better than the synthetic drugs (Koirala et al., 2021; Zou et al., 2020). Therefore, how to develop the food‐derived antihypertensive peptides is of great significance to human health. Previous reports illustrated that oxidative stress might be one of the causes of hypertension (Aondona et al., 2021; Sinha & Dabla, 2015). Therefore, it is necessary to study the effect of WJSPHs on the inhibitory activity of ACE.

**FIGURE 13:**
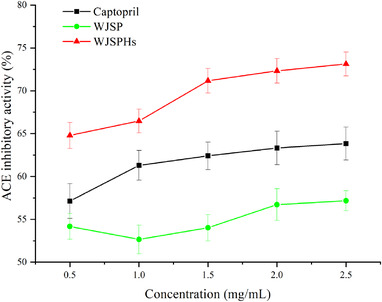
ACE inhibitory activity of WJSP, WJSPHs, and captopril

The ACE inhibitory activities of WJSPHs are shown in Figure 13. It can be seen that the ACE inhibitory capacity was increased obviously with the increase of the concentration of WJSPHs in the concentration range of 0.5−2.5 mg/ml. When the concentration of WJSPHs was 2.5 mg/ml, the ACE inhibition ratio was 73.02%, which shows the best ACE‐inhibition ability than captopril (63.83%), WJSP (57.17%), mung bean protein hydrolysates (71.22%), and wheat bran protein hydrolysates (58.40%) described by Xie et al. (2019) and Zou et al. (2020). Therefore, these results indicate that enzymolysis can significantly increase the ACE inhibitory activity of WJSP, and WJSPHs may be a promising product to treat hypertension.

## 4 CONCLUSION

Our research reported on the physico‐chemical properties, antioxidant activity, and ACE inhibitory activity of WJSP and WJSPHs. Our results indicate that alcalse hydrolysates exhibited higher DH, DPPH radicals‐scavenging activity, and smaller molecular fragment than neutrase and papain hydrolysates. Furthermore, the molecular weight distribution of WJSPHs was found to be mainly under 5,000 Da, and secondary structure of WJSPHs were mainly random coils confirmed by combined CD and FTIR spectra. In addition, WJSPHs showed better thermal stability of 119.29℃ than WJSP of 110.50℃. All these results indicate that WJSPHs possess smaller molecular weight and looser structure, which may enhance the functional properties and biological activities of WJSPHs. On the other hand, WJSPHs show great antioxidant activities including DPPH radical‐scavenging activity (94.60%), ABTS^+^ radical‐scavenging activity (90.84%), superoxide radical‐scavenging activity (44.77%), and hydroxyl radical‐scavenging activity (47.77%). The antioxidant activity test in HepG2 cells showed that WJSPHs decreased the release of ROS and MDA, enhanced the cell viability and the enzyme activity of SOD and GSH‐Px in HepG2 cells induced by H_2_O_2_, to further protect against oxidative stress‐induced damage. Furthermore, WJSPHs exhibited high ACE inhibitory activity (73.20%) in vitro showing great antioxidant activity. Consequently, these results suggest WJSPHs may be a promising antioxidant food to prevent some oxidative‐related diseases in future.

## AUTHOR CONTRIBUTIONS

**Rongxin Han**: Writing—original draft. **Shuai Shao**: Conceptualization; writing—review & editing. **Hongyin Zhang**: Methodology; software. **Hongyu Qi**: Resources; software. **Fengqin Xiao**: Investigation; software. **Yingxin Shen**: Methodology; validation. **Lin Fan**: Methodology; resources. **Haidong Wang**: Investigation. **Daqing Zhao**: Conceptualization; formal analysis; project administration. **Guangzhe Li**: Formal analysis; software. **Mingming Yan**: Writing—Review & editing

## CONFLICT OF INTEREST

The authors declare no conflict of interest.
